# Expression of pathogenesis related genes in response to salicylic acid, methyl jasmonate and 1-aminocyclopropane-1-carboxylic acid in *Malus hupehensis *(Pamp.) Rehd

**DOI:** 10.1186/1756-0500-3-208

**Published:** 2010-07-27

**Authors:** Jiyu Zhang, Xiaoli Du, Qingju Wang, Xiukong Chen, Dong Lv, Kuanyong Xu, Shenchun Qu, Zhen Zhang

**Affiliations:** 1 College of Horticulture, Nanjing Agricultural University, 1 Weigang, Nanjing, Jiangsu, China; 2 Liaoning Agricultural Vocation-Technical College, Yingkou, Liaoning, China

## Abstract

**Background:**

Many studies have been done to find out the molecular mechanism of systemic acquired resistance (SAR) in plants in the past several decades. Numbers of researches have been carried out in the model plants such as arabidopsis, tobacco, rice and so on, however, with little work done in woody plants especially in fruit trees such as apple. Components of the pathway of SAR seem to be extremely conserved in the variety of species. *Malus hupehensis*, which is origin in China, is strong resistance with rootstock. In the study, we attempted to make the expression pattern of pathogenesis related (PR) genes which were downstream components of the SAR pathway in response to salicylic acid(SA), methyl jasmonate(MeJA) and 1-aminocyclopropane-1-carboxylic acid(ACC) in *Malus hupehensis*.

**Findings:**

In order to analyze the expression pattern, the partial sequence of three PR genes from *Malus hupehensis*, *MhPR1*, *MhPR5 *and *MhPR8 *was isolated. These three PR genes were induced by SA, MeJA and ACC. However, *MhPR1*, *MhPR5 *and *MhPR8 *performed a distinct pattern of expression in different plant organs. *MhPR5 *and *MhPR8 *were basal expression in leaves, stems and roots, and *MhPR1 *was basal expression only in stems. The expression of *MhPR1*, *MhPR5 *and *MhPR8 *was enhanced during the first 48 h post-induced with SA, MeJA and ACC.

**Conclusions:**

The results showed that a distinct pattern of expression of PR genes in *Malus hupehensis *which differed from the previous reports on model plants arabidopsis, tobacco and rice. *MhPR1*, *MhPR5 *and *MhPR8 *were induced by SA, MeJA and ACC, which were regarded as the marker genes in the SAR response in *Malus hupehensis*. In contrast with herbal plants, there could be specific signal pathway in response to SA, JA and ET for woody plants.

## Background

In nature, basal defenses have been evolved by plants against the adverse conditions such as pathogens, insects, and injuries and so on. Plants have been invaded multiple aggressors simultaneously or subsequently, which could affect the principal induced defense response of the host plants [1]. Botanists have acknowledged that the phytohormones salicylic acid (SA), jasmonic acid (JA), and ethylene (ET) play key roles in the signaling network that regulates the induced defense responses in plants [2-6]. Plants have evolved powerful regulatory potential by Cross talk among SA-, JA-, and ET-dependent signaling pathways to effectively and efficiently adapt to the complex hostile situation [6-8]. SA-, JA-, and ET-dependent pathways regulated defense responses and were differentially effective to against specific types of invaders in plants [9,10].

Pathogenesis-related (PR) proteins, which are the downstream components of systemic acquired resistance (SAR) in plants, have been used routinely for the defense status of plants with positive antimicrobial activity. PR-proteins are induced in response to attack by pathogens [11]. Plants are able to coordinate the expression of specific PR genes in response to attack by relevant pathogens at the molecular level.

Regard of SAR and PR genes, there is plenty of information chiefly related to model plants, such as arabidopsis [12], tomato and tobacco [13,14]. Inductions of *PR*1, 5, and 8 are characteristic of SAR in several herbaceous plants. *PR-8 *is strongly induced in cucumber by SA, but less INA (2, 6-dichloroisonicotinic acid) [15]. However, there was lest of the work done in woody plants especially in fruit trees such as apple. Identified from apple, *PR-2*, *PR-5 *and *PR-8 *are induced in response to inoculation with the apple pathogen, *Erwinia amylovora*, but they are not induced in young apple stems by treatment with elicitors of SAR in herbaceous plants [16]. However, little work had been done on the expression pattern of pathogen protein genes in response to SA, MeJA and ET in woody perennials.

*Malus hupehensis*, which was origin in China, was strong resistance to rootstock. In this study, we assayed the expression of *PR *genes in *Malus hupehensis *seedlings tissue following treatment with SA, methyl jasmonate (MeJA) and ET prescursor 1-aminocyclopropane-1-carboxylic acid (ACC), including in leaves, stems and roots.

## Material and methods

### Plant material and treatment

*Malus hupehensis *(Pamp.) Rehd. tissue culture seedlings were favored by Yujin Hao professor of Shandong agricultural university and subcultured in Murashige and Skoog (MS) medium supplied with 6-BA(0.5 mg/L)and NAA(0.1 mg/L)cultured under a 16 h-light (25°C)/8 h-dark (25°C) cycle. Seedlings were rooted in 1/2 MS supplied with 0.1 mg/L NAA after three weeks. *Escherichia coli *strain DH5a cells were used for the cloning of the *MhPR1*, *MhPR5*, *MhPR8 *genes and the *Mhtubulin *was regarded as housekeeping gene in semi-quantitative RT-PCR assay.

*Malus hupehensis *culture seedlings rooted three weeks were sprayed with 0.1 mM salicylic acid (SA) (Sigma), 0.02 mM MeJA (Sigma), 0.01 mM ACC (Sigma) supplied with 0.015% (v/v) Silwet L77 (Van Meeuwen Chemicals) respectively for 4,12 and 48 h, taking the seedlings without treatments as control. Three seedlings were duplicated for each treat. The leaves, stems and roots were frozen by plunging the excised portions into liquid nitrogen. After that, the tissues were stored at -70°C for further research in future.

### Isolation of total RNA and first strand cDNA Synthesis

Total RNA was isolated as described previously by CAI et al. [17]. Genomic DNA of total RNA was eliminated by treating with RNase-free DNase I (TaKaRa, Code No: D2215) according to the manufacturer's instruction. The total RNA (1 μg) was reversely transcribed with the ReverTra Ace qPCR RT Kit for the cDNA synthesis according to the instructions of manufacturer (TOYOBO, Code No.: FSQ-101). First strand cDNA samples were diluted 1:10 with sterile double distill water and stored at -20°C before being used as template in semi-quantitative RT-PCR.

### Semi-quantitative RT-PCR Analysis

Primers (Table 1) were designed based on the known *PR *genes sequences deposited in GenBank and were used to amplify the putative *PR *genes and the housekeeping gene *tubulin *fragments from cDNA of *Malus hupehensis *were used as an internal standard. The amplicons of PCR for *PR*-1, *PR*-5, *PR-*8 and *Tubulin *were cloned through PMD18-T vector and sequenced at Shanghai Invitrogen Biotechnology Co., Ltd. The nucleotide sequences were compared with those released in GenBank Accession Nos. using the BLASTn program [18].

**Table 1:** Primers used for semi-quantitative RT-PCR

name	Forward Primer Sequence [5'-3']	Reverse Primer Sequence [5'-3']	Ampicon size
PR1	CCACAAAGAGAACAAACCATTAAC	GACCAACGCCTACTGCTG	160
PR5	CATGTCCTCCCACAGAGTAC	ATATAATCCCATTTCGTGCTTATG	153
PR8	AGCAGGTTCTATGACAACGG	CGCCATCATCCCTAACACAC	112
tubulin	AGGATGCTACAGCCGATGAG	GCCGAAGAACTGACGAGAATC	192

The primers were set using the software beacon designer 7.0 and were synthesized at Shanghai Invitrogen Biotechnology Co., Ltd.

Semi-quantitative RT-PCR amplification was carried out on Alpha Unit Block Assembly for DNA Engine Systems (BIO-RAD) with 20 μL of reaction solution, containing 1 μL of 10-fold-diluted cDNA, 0.3 μL 10 pM of each primer (invitrogen), 1.6 μL dNTP (TaKaRa Code: D4030A), 2 μL 10 × PCR buffer, 1.5 μL MgCl_2_, 0.125 μL rTaq enzyme (TaKaRa Code:R10T1 M ), and 13.175 μL sterile double distill water. The reaction protocol as the follows: initial denaturation step at 94°C for 3 min followed by 35 cycles at 94 °C for 30 s, 57°C for all the primer sets for 30 s, 72°C for 30 s, exception 25 cycles for *Mhtubulin*, and a final elongation step at 72°C for 5 min. 10 μL of PCR products were separated by electrophoresis in 1.5% agarose gels and visualized under UV light after staining with ethidium bromide.

## Results

### Identification of *MhPR1*, *MhPR5*, *MhPR8 *and *MhTubulin *from *Malus hupehensis*

In order to analyze the expression pattern of *PR *genes in response to SA, MeJA and ACC in *Malus hupehensis*, the partial sequence of *MhPR1*, *MhPR5 *and *MhPR8 *were isolated from *Malus hupehensis *and the corresponding primes were set according to the sequence of  PR genes isolated from apple (*Malus *× *domestica *cv.) of which NCBI GenBank accession numbers were DQ318212, DQ318213 and DQ318214 respectively [19] (Table 2). The nucleotide sequences of *MhPR1*, *MhPR5 *and *MhPR8 *cloned from *Malus hupehensis *had 96%, 98% and 99% identities with DQ318212, DQ318213 and DQ318214 respectively, and the corresponding sequences' numbers in GenBank were GU317941, GU317942 and GU317943.

**Table 2:** Side-by-side comparison of partial sequences of three PR genes from *Malus hupehensis *with their respective PR genes from apple (*Malus *× *domestica *cv.)

	*MhPR1*	*MdPR1*	*MhPR5*	*MdPR5*	*MhPR8*	*MdPR8*
Identities	155/160 (96%)		151/153 (98%)		111/112 (99%)	
Expect	2e-68		7e-69		6e-48	
Gaps	0%		0%		0%	
GenBank	GU317941	DQ318212	GU317942	DQ318213	GU317943	DQ318214

Considered as the housekeeping gene, the *MhTubulin *gene was isolated from *Malus hupehensis *and has 99% nucleotide sequence identities with TC31643 [20] and the nucleotide sequence of *Malus hupehensis Tubulin *gene was deposited in GenBank with corresponding accession number GU317944.

### Pathogenesis related (PR) genes were induced by SA, MeJA and ACC in leaves

**Figure 1:**
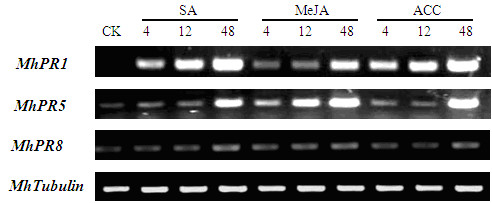
**PR genes were induced by SA, MeJA and ACC in leaves**. PR genes were induced by SA, MeJA and ACC in leaves using semi-quantitative RT-PCR. *MhTubulin *transcript levels were used to normalize the samples. There were 25 cycles for *MhTubulin *and 35 for *MhPR *genes. 10 μl RT-PCR production was assayed by electrophoresis on ethidium bromide stained 2.0% agarose gels. *Mh*: *Malus hupehensis*. PR1: pathogenesis related protein 1; PR5: pathogenesis related protein 5; PR8: pathogenesis related protein 8.

Taking the *MhTubulin *as housekeeping gene, semi-quantitative RT-PCR analysis was performed with the RNA isolated from leaves of *Malus hupehensis *tissue culture seedlings following the treatments with SA, MeJA and ACC (Figure 1). *MhPR5 *and *MhPR8 *were basal expression in leaves, however, *MhPR1 *was not. Expression levels of *MhPR1*, *MhPR5 *and *MhPR8 *were enhanced after inducing by SA, MeJA and ACC. During the first 48 h, the expression of *MhPR1 *was strongly induced at 4 h and gradually increased after sparing with SA and ACC, and was weakly induced at 4 h and 12 h, and was strongly induced at 48 h in response to MeJA. *MhPR5 *was also strongly induced by SA at ACC at 48 h post-induced and by MeJA at 12 h post-induced. *MhPR8 *was delicately induced by SA, MeJA and ACC.

### Pathogenesis related genes were induced by SA, MeJA and ACC in stems

**Figure 2:**
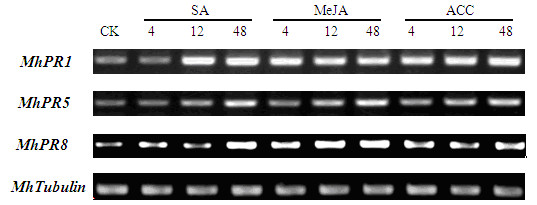
**PR genes were induced by SA, MeJA and ACC in stems using semi-quantitative RT-PCR**. PR genes were induced by SA, MeJA and ACC in stems using semi-quantitative RT-PCR. *MhTubulin *transcript levels were used to normalize the samples. There were 25 cycles for *MhTubulin *and 35 for *MhPR *genes. 10 μl RT-PCR production was assayed by electrophoresis on ethidium bromide stained 2.0% agarose gels. *Mh*: *Malus hupehensis*. PR1: pathogenesis related protein 1; PR5: pathogenesis related protein 5; PR8: pathogenesis related protein 8.

The semi-quantitative RT-PCR demonstrated that all the three identified *PR *genes were induced during 48 h following the treatments with SA, MeJA and ACC in stems (Figure 2). *MhPR1*, *MhPR5 *and *MhPR8 *were basal expression in stems. In contrast, for leaves, *MhPR1 *gene was intensively induced at 4 h post-induced by MeJA and ACC and at 12 h by SA. *MhPR5 *was delicate induced at 12 h and strongly induced at 48 h post-induced by SA, MeJA and ACC. Enhance expression of *MhPR8 *was found at 4 h after induced by SA, MeJA and ACC.

### Pathogenesis related genes were induced by SA, MeJA and ACC in roots

**Figure 3:**
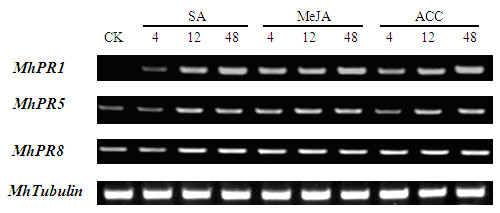
**PR genes were induced by SA, MeJA and ACC in roots through semi-quantitative RT-PCR**. PR genes were induced by SA, MeJA and ACC in roots using semi-quantitative RT-PCR. *MhTubulin *transcript levels were used to normalize the samples. There were 25 cycles for *MhTubulin *and 35 for *MhPR *genes. 10 μl RT-PCR production was assayed by electrophoresis on ethidium bromide stained 2.0% agarose gels. *Mh*: *Malus hupehensis*. PR1: pathogenesis related protein 1; PR5: pathogenesis related protein 5; PR8: pathogenesis related protein 8.

The semi-quantitative RT-PCR assay showed that *MhPR1*, *MhPR5 *and *MhPR8 *were induced by SA, MeJA and ACC in roots. Similar as in leaves, *MhPR5 *and *MhPR8 *were basal expression in roots, in which *MhPR1 *was not. Enhanced expression of *MhPR1 *was conspicuous at 4 h, and the expression level was gradually increasing after the treatment of SA, MeJA and ACC in roots. However, *MhPR5 *and *MhPR8 *were weakly induced during the first 48 h after processed with SA, MeJA and ACC (Figure 3).

## Discussion

In 1970, PR proteins were founded in genotypes of tobacco infected with tobacco mosaic virus (TMV) [21]. After that, numbers of PR proteins had been reported as discovering in a wide variety of plant species [22], such as *PR1 *(unknown), *PR2 *(β-1,3-glu*c*anase), *PR3 *(chitinase type I, II, IV, V, VI, VII), *PR5*(osmotins), *PR8*(chitinase type III )and *PR10 *and so on[23,24]. In woody plants, the cDNA sequences of *PR1 *(GeneBank accession: AF195236, AF195237, FJ594483) and *PR5 *(thaumatin-like protein, GeneBank accession: FJ197337, FJ795347) were isolated from *Pyrus*. PR1 (*PR1a*, *PR1b*, *PR1c*), *PR-2*, *PR-5 *and *PR-8 *were identified as candidates in the responses to the attack by *E. amylovora *of apple based on the similarity to genes documented as involved in SAR in other plants. They were up-regulated in response to inoculation with the pathogen, *E. amylovora*[16]. In the study, the complete cDNA sequences of *PR2 *(β-1, 3-*glucanase*) and *PR3 *(*chitinase*) (date not shown) were cloned, as well as the partial cDNA sequence of *PR1*, *PR5 *and *PR8 *from *malus hupehensis*.

In *Arabidopsis*, *PR1 *is upregulated only by SA or INA (2, 6-dichloroisonicotinic acid), but is not induced by MeJA or ET [12]. In tobacco, *PR1 *is not only induced by SA but also recognized to be induced by a combination of ethylene and MeJA [25]. However, *PR1 *was not induced by MeJA or ET. *PR1b *gene was induced weakly by SA, but was strongly activated by exogenous JA in rice [26]. The expression of a member of the *PR1 *family was found to be constitutive and unaffected by treatments with BTH or salicylic acid in pear plants. The marker gene *PR1 *is clearly not concerned first and foremost in the SAR response in pear [27]. In our study, it was surprised that the *MhPR1 *gene was not only strongly induced by SA but also intensively upregulated by MeJA and ACC in leaves, stems and roots in *Malus hupehensis *through semi-quantitative RT-PCR amplification. This conclusion suggested that *MhPR1 *might be a distinct pattern of expression differing from the reports previously for herbage plants such as arabidopsis, tobacco and rice. The result showed the *MhPR1 *gene could be regarded as a marker gene in the SAR response in *Malus hupehensis*.

In tobacco, *PR5 *gene is regulated by SA, MeJA, ET, abscisic acid (ABA) and so on [25,28-30]. The combination of ET and MeJA induced both mRNA and protein of *PR5 *to accumulate in tobacco [25]. The expression of *PR5 *was in response to SA and INA in arabidopsis. *MhPR5 *gene was strongly induced by the treatment with SA, MeJA and ACC in leaves, and weakly upregulated in stems and roots. The same as *MhPR1*, *MhPR5 *gene was regarded as a marker gene in the SAR response in *Malus hupehensis*. Expression pattern of *MhPR8 *was different in all kinds of tissues in the study. *MhPR8 *was strongly induced in stems, weakly induced in leaves and roots with SA, MeJA and ACC.

PR genes have a distinctive pattern of expression in *Malus hupehensis *in contrast with the Arabidopsis, tobacco and rice. It was surprised that *MhPR1*, *MhPR5 *and *MhPR8 *expression enhanced in response to SA, MeJA and ACC in leaves, stems and roots. Thus, the results indicated that more than one single signal pathway regulated one member of the PR genes together and a signal pathway could regulate some members of the PR genes at the same time. Signal pathways of resistant to pathogen of woody fruit trees are different from herbage plants. The further study on the SA-, JA-, and ET-dependent signaling pathways response to pathogen in woody plants should be necessary.

## Conclusions

The expression of *MhPR1*, *MhPR5 *and *MhPR8 *were enhanced during the first 48 hours after induced with SA, MeJA and ACC in leaves, stems and roots. Thus, *MhPR1*, *MhPR5 *and *MhPR8 *genes were regarded as the marker genes in the SAR response in *Malus hupehensis*. In contrast with herbage plants, *PR *genes have a distinctive expression pattern in response to SA, MeJA and ACC in woody plants. It was feasible that the expression pattern of PR genes in woody plants attacked by pathogen could be different with the herbage plants. The woody plants could have specific signal pathway in response to pathogen.

## Competing interests

The authors declare that they have no competing interests.

## Authors' contributions

JZ carried out all the experiments, prepared the figures and drafted the manuscript. XD, DL and XC performed the sample preparation and participated in tables drawing. KX and QW assisted in manuscript revising and provided helpful discussions. SQ and ZZ were responsible for experimental design and revised and polished the manuscript. All authors have read and approved the final manuscript.
